# Supplementary material for: Synthesis of Co-Ni Alloy Particles with the Structure of a Solid Substitution Solution by Precipitation in a Supercritical Carbon Dioxide
Source: Nanomaterials (Basel). 2022 Dec 7;12(24):4366. doi: 10.3390/nano12244366 (PMC9782632; doi:10.3390/nano12244366)
Supplement: Supplementary file 1 [file nanomaterials-12-04366-s001.zip › nanomaterials-2035174-supplementary.pdf]

Supplementary Materials

Synthesis of Co-Ni Alloy Particles with the Structure of a Solid Substitution Solution by Precipitation in a Supercritical Carbon Dioxide

Nikolay Nesterov [1], Vera Pakharukova [1], Svetlana Cherepanova [1], Stanislav Yakushkin [1], Evgeniy Gerasimov [1], Dmitry Balaev [2,3], Sergei Semenov [2,3], Andrey Dubrovskii [2,3] and Oleg Martyanov [1,*]

[1] Boreskov Institute of Catalysis, Siberian Branch, Russian Academy of Sciences, 630090 Novosibirsk, Russia
[2] Kirensky Institute of Physics, Krasnoyarsk Scientific Center, Siberian Branch, Russian Academy of Sciences, 660036 Krasnoyarsk, Russia;
[3] Siberian Federal University, Institute of Engineering Physics and Radioelectronics, 660041 Krasnoyarsk, Russia
* Correspondence: oleg@catalysis.ru

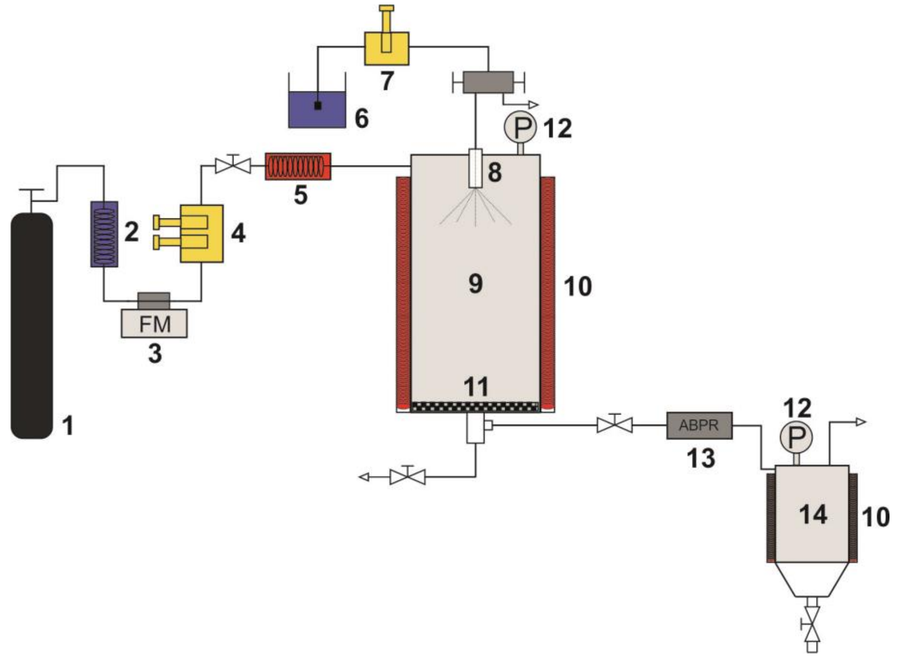

Figure S1. The basic scheme of the anti-solvent setup SAS-50: 1 – CO_2 tank, 2 – CO_2 cooler, 3 – CO_2 mass flowmeter, 4 – CO_2 high-pressure pump, 5 – CO_2 preheater, 6 – precursor solution, 7 – high-pressure precursor solution, 8 – nozzle, 9 – precipitation vessel, 10 – heating jacket, 11 – metal filter, 12 – manometer, 13 – automatic back-pressure regulator, 14 – separator.

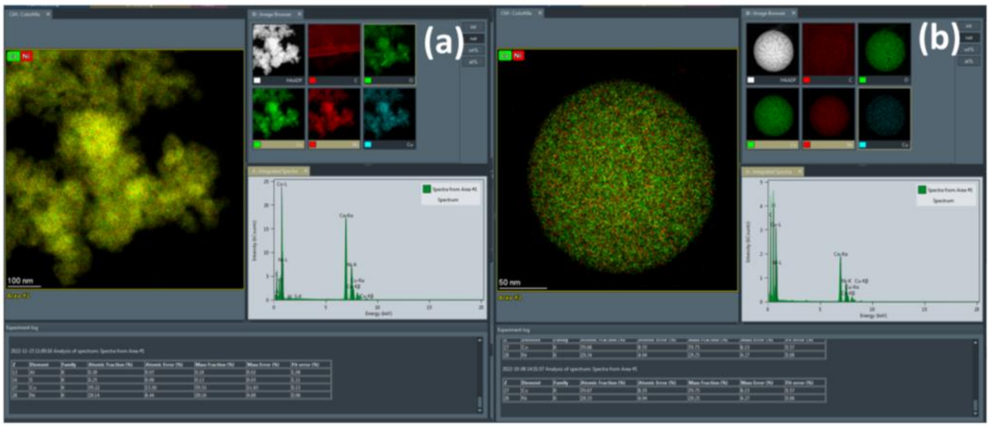

Figure S2. EDX data of reduced samples: Co2Ni1_W0 – (a); Co2Ni1_W8 – (b).

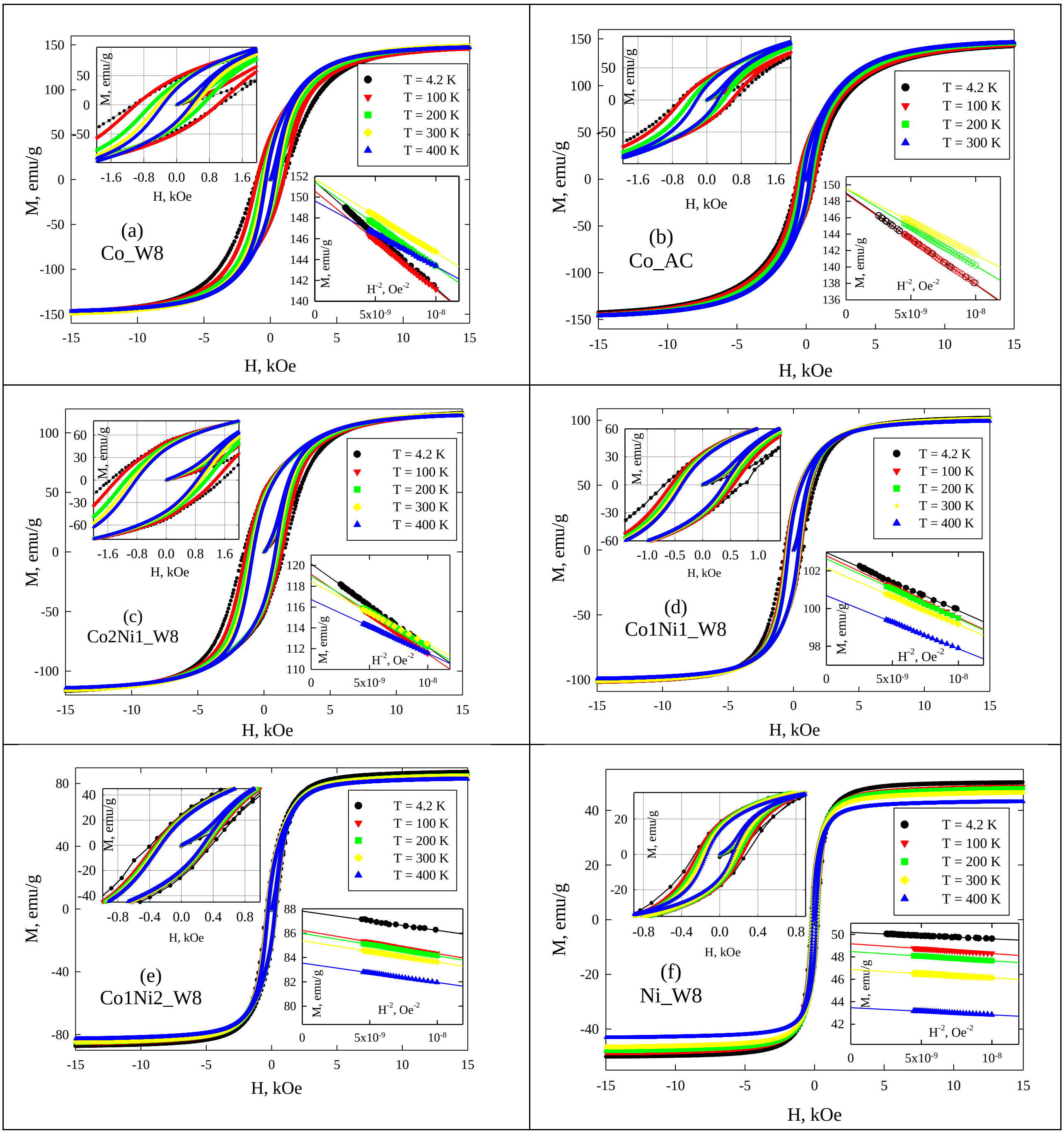

Figure S3. M(H) hysteresis loops at 300 K for all samples . Left insertion – M(H) near the origin; right insertion – high-field magnetization behavior in 1/H^2 coordinates for the fields > 10 kOe, with linear regression. Registration temperature is shown on the figure.

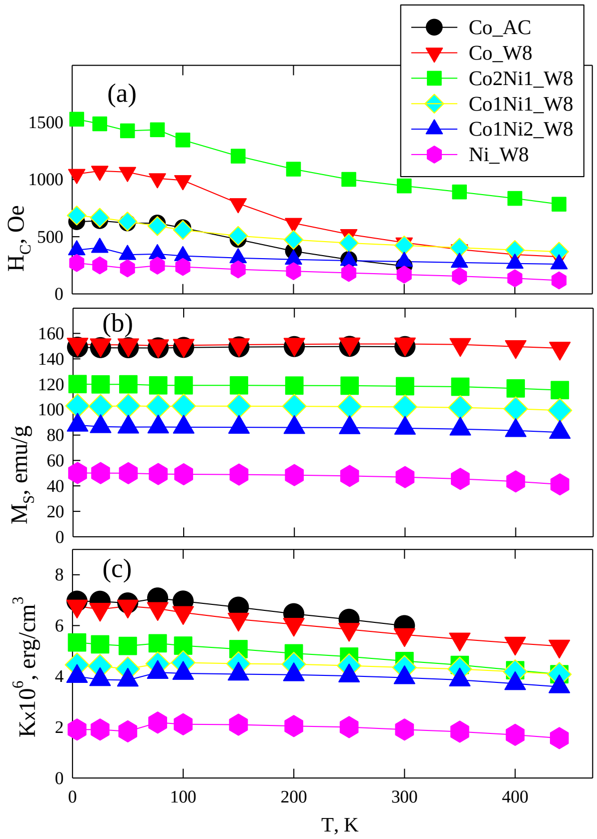

Figure S4. Temperature dependences of coercivity $H_C(T)$ – (a), saturation magnetization M_S – (b), and magnetic anisotropy constant K for studied series of samples. M_S and K were obtained from high-field M(H) data – right insets of figure.S3 by the law (1). Lines are guides for "eye".

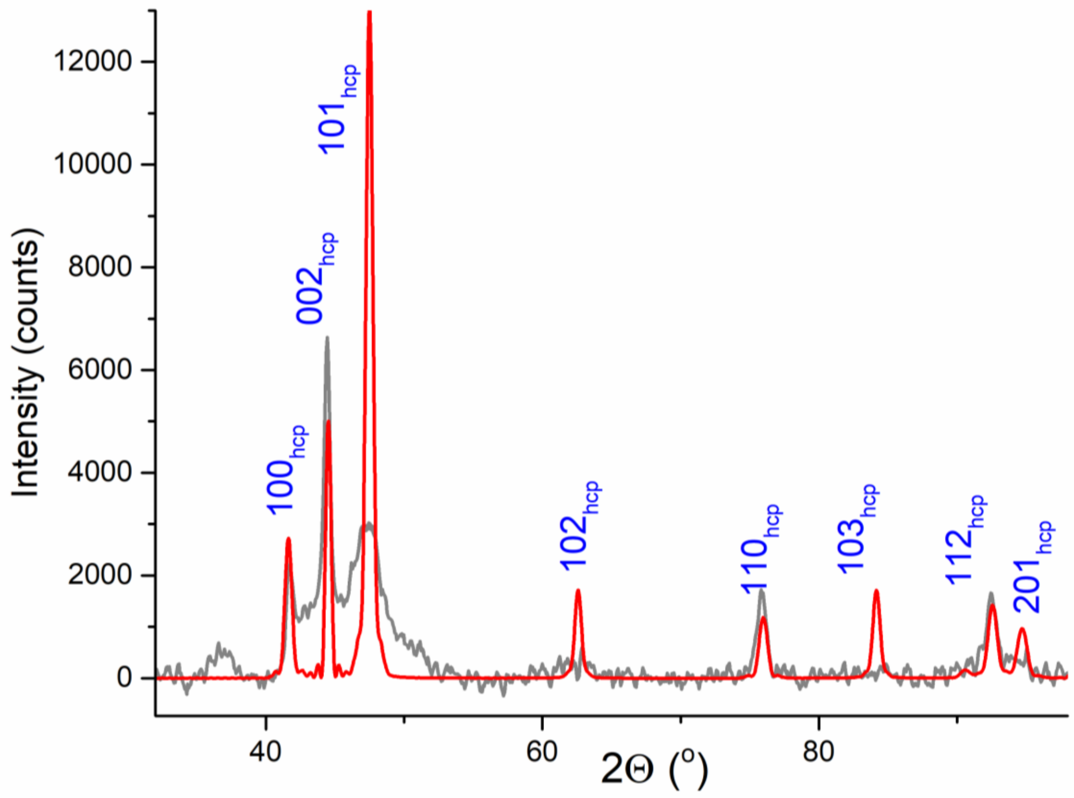

Figure S5. Experimental XRD pattern (grey curve) for Co_W8 sample and calculated XRD profile (red curve) for model of Co⁰ crystallite with defect-free hcp structure.

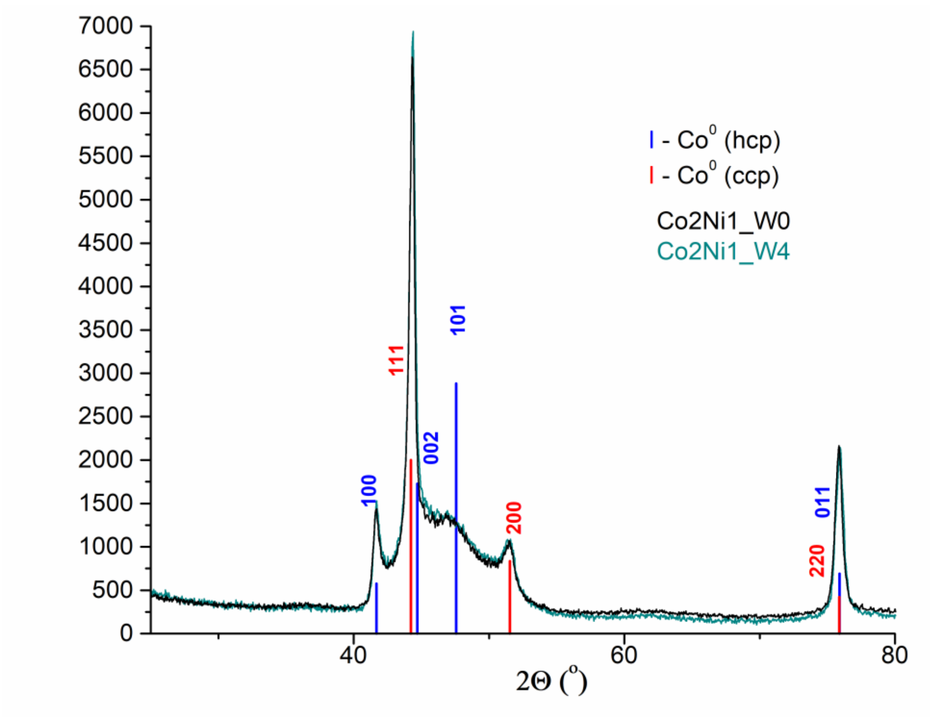

Figure S6. XRD patterns of obtained Co2Ni1_W0 and Co2Ni1_W4 samples.

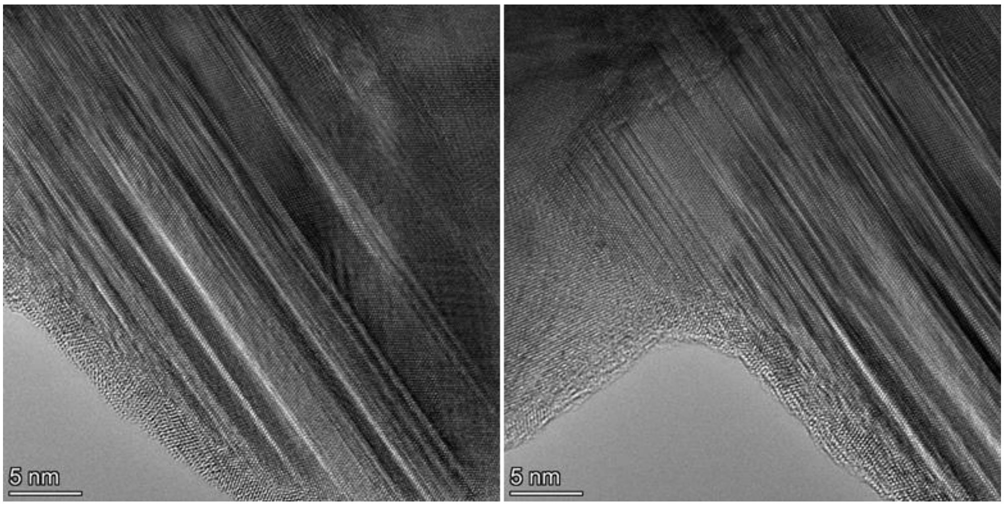

Figure S7. HRTEM image of nanostructured metallic nanoparticles in Co_2Ni_W0 sample with thick (left) and thin (right) alternating domains with different structures.
